# Editorial: Small organic molecules with anticancer activity

**DOI:** 10.3389/fchem.2023.1254312

**Published:** 2023-08-23

**Authors:** A. F. M. Motiur Rahman, Mohammad Sayed Alam, Youngjoo Kwon

**Affiliations:** ^1^ Department of Pharmaceutical Chemistry, College of Pharmacy, King Saud University, Riyadh, Saudi Arabia; ^2^ Department of Chemistry, Jagannath University, Dhaka, Bangladesh; ^3^ College of Pharmacy, Graduate School of Pharmaceutical Sciences, Ewha Womans University, Seoul, Republic of Korea

**Keywords:** small organic molecule, anticancer drug, cytotoxicity, pyrazolyl-s-triazine, sulfonate tetrandrine, spermidine

Cancer is a complex disease comprising nearly a hundred types, posing a significant threat to global public health. According to the WHO’s report, the most common types are breast, lung, colon and rectum, prostate, skin and stomach cancer, while most common causes of cancer death are lung, colon and rectum, liver, stomach and breast cancer. Although cancer is often observed in middle-aged to older adults, it can affect individuals of any age. In the world, more than ten million lives are lost to cancer each year. Despite the availability of numerous anti-cancer drugs, the discovery of potential agents remains crucial to realizing a cure for this debilitating disease. Small organic molecules have received significant attention from researchers in the field of medicinal chemistry in recent decades as potential anticancer agents due to their pharmacokinetic and pharmacodynamic properties, such as i) size (molecular weight less than 500 Da), ii) specificity (interact with specific molecular targets, such as enzymes, receptors, and transporters, with high specificity), iii) stability (small organic molecules are often more stable than larger molecules and can withstand harsh conditions, such as high temperatures and acidic or basic environments), iv) synthesis (small organic molecules can be synthesized in large quantities using chemical synthesis, which can be more cost-effective than producing biologics), v) oral bioavailability (many small organic molecules are orally bioavailable, meaning they can be taken as pills or capsules rather than requiring injection or infusion). Small molecule anticancer drugs are classified into different categories based on their mode of action, with kinase inhibitors being the largest family. While many small molecule anticancer drugs have been approved for clinical use, the development of new and multitargeting anticancer agents remains a critical necessity. This editorial also focuses on the potential of small organic molecules as anticancer agents, as well as the challenges and opportunities associated with their development and clinical use. The present edition of Frontiers in Chemistry highlights small organic molecules with anticancer activity, offering insight into the synthesis of novel anticancer agents and their mode of action.

In this Research Topic, two research and one review article were published. In the first research article, Shawish et al. designed and synthesized a series of pyrazolyl-*s*-triazine compounds with an indole motif, and their potential as anticancer agents targeting dual EGFR and CDK-2 inhibitors were evaluated. Several of the compounds showed promising cytotoxic activity against two cancer cell lines, A549 and MCF-7, while exhibiting a superior safety profile compared to a reference drug in non-cancerous human dermal fibroblasts. Notably, one of the compounds, namely, 4-(5-amino-3-(1*H*-indol-3-yl)-1*H*-pyrazol-1-yl)-*N*-(4-chlorophenyl)-6-morpholino-1,3,5-triazin-2-amine, demonstrated potent EGFR inhibition and caused significant apoptosis in lung cancer cells. These findings suggest that the synthesized compounds could be considered as lead candidates for further research into target-oriented anticancer agents. Ultimately, this study could lead to the development of new and effective treatments for cancer.

In the second research article, Jiang et al. synthesized a series of C14 sulfonate tetrandrine derivatives and evaluated their potential as chemotherapeutic agents for hepatocellular carcinoma (HCC). Most of the synthesized compounds demonstrated greater antiproliferative activity against four different HCC cell lines than the lead compound, tetrandrine. A compound, namely, 14-*O*-(5-chlorothiophene-2-sulfonyl)-tetrandrine, showed the strongest antiproliferative effect and was found to induce apoptosis in HCC cells via a mitochondria-mediated intrinsic pathway. Additionally, above compound also inhibited HCC cell proliferation, migration, and invasion, suggesting its potential as a candidate for anti-HCC therapy. This study highlights the potential of structural modifications of natural compounds, like tetrandrine, in the development of novel chemotherapeutic agents for the treatment of HCC, which could ultimately lead to improved patient outcomes.

This review article reported by Prasher et al. focuses on the anticancer properties of spermidine, a naturally occurring polyamine compound found in semen and plant sources. The authors discuss the molecular mechanisms by which spermidine halts the cell cycle, inhibits tumor cell proliferation, and suppresses tumor growth. They also explore how spermidine triggers autophagy by regulating key oncologic pathways and how chemically modified derivatives of spermidine hold great potential for prognostic, diagnostic, and therapeutic applications against various malignancies. In addition to its anticancer properties, spermidine has also shown to have neuroprotective, anti-aging, and anti-inflammatory effects. The authors highlight the potential of spermidine as a target for the development of new anticancer chemotherapeutics and emphasize the importance of further clinical translation of spermidine and its analogues for the advancement of cancer medications. Overall, this review provides valuable insights into the molecular mechanisms and therapeutic potential of spermidine in cancer management.

Taken together, the three articles in this Research Topic highlight the promising potential of chemical biology in the development of novel and effective treatments for cancer and hepatocellular carcinoma, as well as the importance of exploring natural compounds and their derivatives in cancer management. These findings provide valuable insights into the molecular mechanisms underlying cancer pathogenesis and the potential of chemical modifications to improve the efficacy and safety of anticancer drugs.

